# Author Correction: Acute thiamethoxam toxicity in honeybees is not enhanced by common fungicide and herbicide and lacks stress-induced changes in mRNA splicing

**DOI:** 10.1038/s41598-021-98193-4

**Published:** 2021-09-21

**Authors:** Pâmela Decio, Pinar Ustaoglu, Thaisa C. Roat, Osmar Malaspina, Jean-Marc Devaud, Reinhard Stöger, Matthias Soller

**Affiliations:** 1 Universidade Estadual Paulista (UNESP), Instituto de Biociências, Centro de Estudos de Insetos Sociais, Rio Claro, São Paulo, Brazil; 2 School of Biosciences, College of Life and Environmental Sciences, University of Birmingham, Edgbaston, Birmingham, B15 2TT UK; 3 Research Center On Animal Cognition, Center for Integrative Biology, Toulouse University, CNRS, UPS, Toulouse, France; 4 School of Biosciences, University of Nottingham, Nottingham/Sutton Bonington Campus, LE12 5RD UK; 5 Present Address: MRC Centre for Molecular Bacteriology and Infection, and Department of Life Sciences, Imperial College London, Ground Floor, Flowers Building, South Kensington Campus, London, SW7 2AZ UK

Correction to: *Scientific Reports* 10.1038/s41598-019-55534-8, published online 16 December 2019

The original version of this Article contained an error in Figure 5A, where the gene structure was incorrectly labelled. The original Figure 5 and accompanying legend appear below.

**Figure 5:**
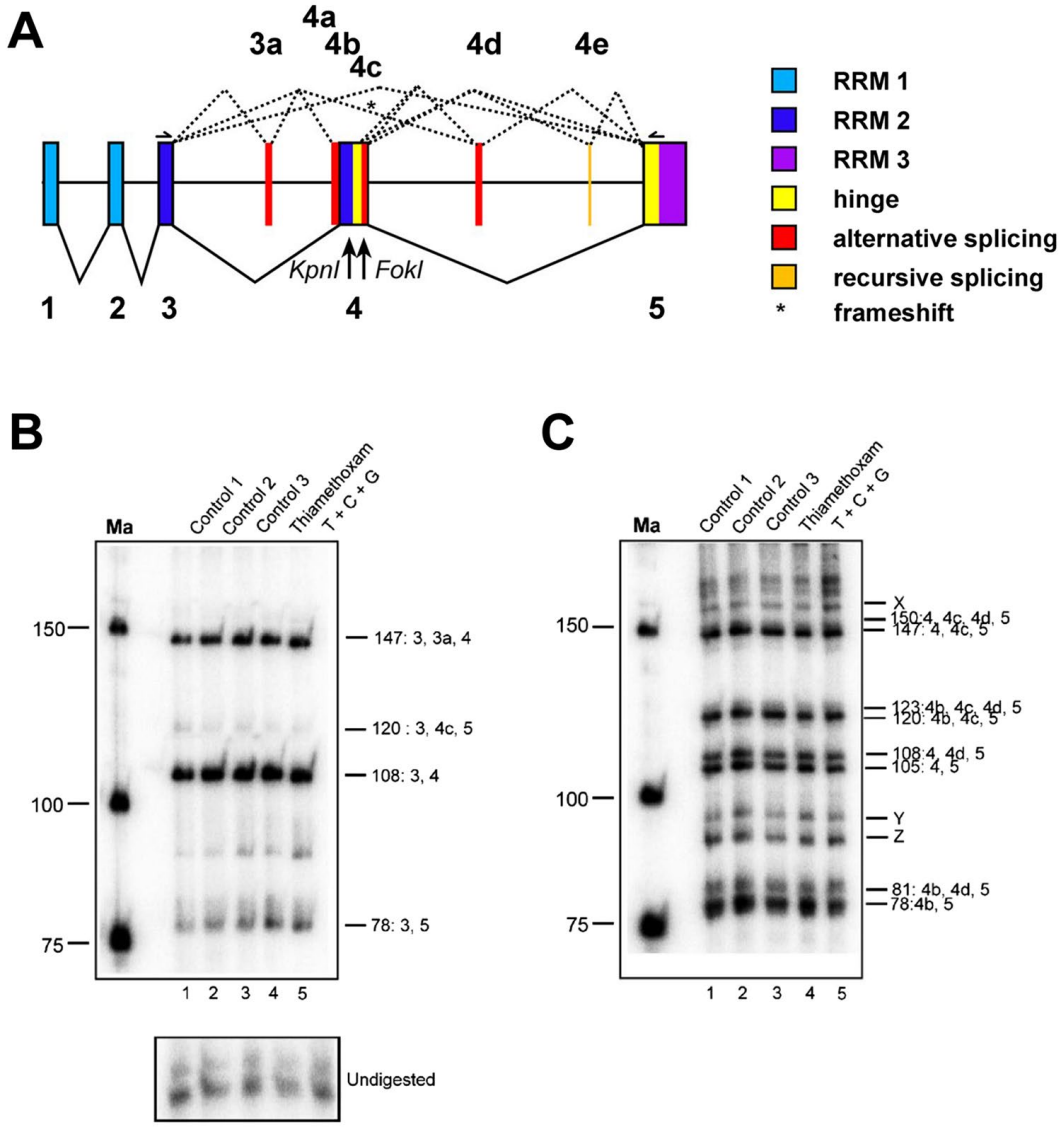
*Apis mellifera elav* alternative splicing in brains of worker bees is unaffected by thiamethoxan, carbendazim and glyphosate. (**A**) Gene structure of *Apis mellifera elav* depicting color-coded functional protein domains with constant exons (1–5, bottom, solid lines) and alternative splicing exons (3a and 4a–d, top, dashed lines). RNA Recognition Motiv 1 (RRM1): light blue, RRM2: dark blue, RRM3: purple, hinge region: red and alternatively spliced parts in red. *Kpn*I and *Fok*I restriction sites used to separate isoforms are indicated below the gene model. An asterisk indicates isoforms that encode truncated proteins by introducing a frameshift. (**B**,**C**) Denaturing polyacrylamide gels (6%) showing the alternative splicing pattern of *elav* by digestion of a 5′ (**B**) or 3′ (**C**) ^32^P labeled RT-PCR product with *Kpn*I (**B**) and *Fok*I (**C**) in control bees dissected immediately after collection (Control 1), control bees fed with water and sucrose for 24 h (Control 2) and control bees injected with water (Control 3) compared to bees injected with thiamethoxam (1 µM) and bees injected with a mixture of thiamethoxam (1 µM, T), carbendazim (2 mM, C) and glyphosate (32 mM, G) 24 h prior dissection. Samples were run on 6% polyacrylamide gel. Ma: DNA marker. The undigested PCR product is shown at the bottom.

The original Article has been corrected.

