# Supplementary material for: Acute thiamethoxam toxicity in honeybees is not enhanced by common fungicide and herbicide and lacks stress-induced changes in mRNA splicing
Source: Sci Rep. 2019 Dec 16;9:19196. doi: 10.1038/s41598-019-55534-8 (PMC6915785; doi:10.1038/s41598-019-55534-8)

Supplementary Figures:

Supplementary Figs 1 and 2, and pictures of uncropped gels

Acute Thiamethoxam toxicity in honeybees is not enhanced by common fungicide and herbicide and lacks stress-induced changes in mRNA splicing

Pâmela Decio[1*], Pinar Ustaoglu[2*]†, Thaisa C. Roat[1], Osmar Malaspina[1], Jean-Marc Devaud[3], Reinhard Stöger[4, 5] and Matthias Soller[2, 5]

[1]Universidade Estadual Paulista (UNESP), Instituto de Biociências, Centro de Estudos de Insetos Sociais, Rio Claro, São Paulo, Brazil.

[2]School of Biosciences, College of Life and Environmental Sciences, University of Birmingham, Edgbaston, Birmingham, B15 2TT, United Kingdom

[3]Research Center on Animal Cognition, Center for Integrative Biology, Toulouse University, CNRS, UPS, Toulouse, France.

[4]School of Biosciences, University of Nottingham, LE12 5RD, United Kingdom

Running title: Neonicotinoid toxicity is not enhanced by common fungicide and herbicide

Key Words: Neonicotinoid, carbendazim, glyphosate, *Apis mellifera*, alternative splicing, *Dscam*, ELAV, RNA binding proteins

[5]Corresponding authors: m.soller@bham.ac.uk, reinhard.stoger@nottingham.ac.uk

* equal contributing authors

Present address: † MRC Centre for Molecular Bacteriology and Infection, and Department of Life Sciences, Imperial College London, Ground Floor, Flowers Building, South Kensington Campus, London SW7 2AZ, UK

Suppl Figure 1

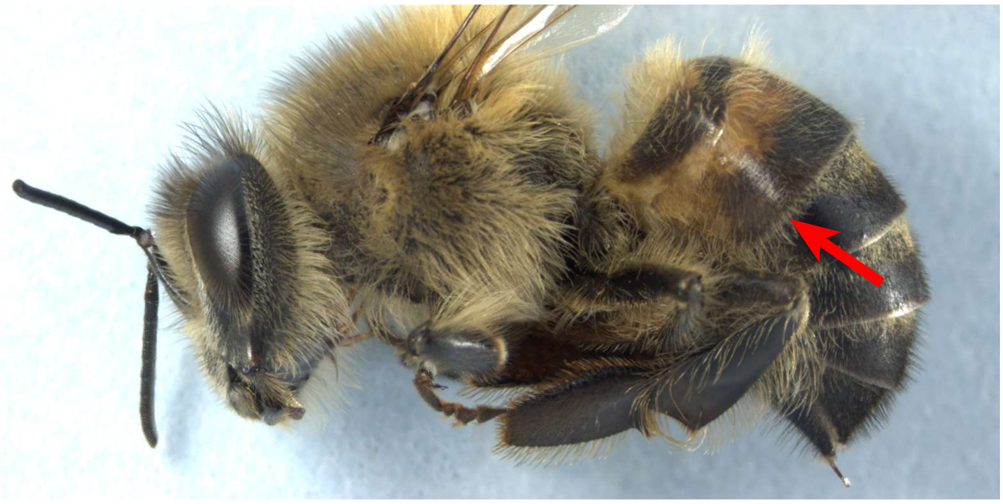

Supplemental Figure 1: Picture of a honey bee. The red arrow points towards the sitre of injection.

Suppl Figure 2

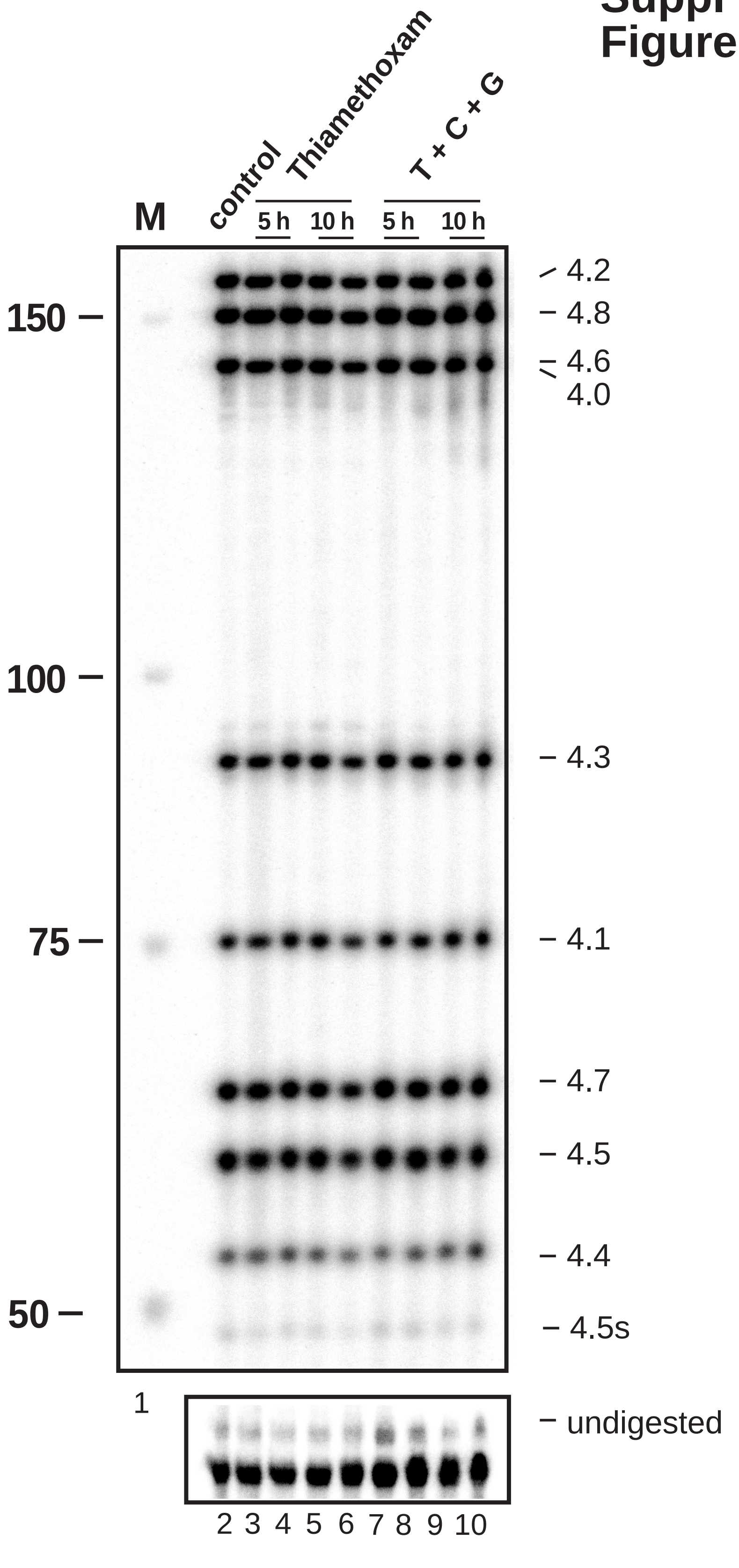

Supplemental Figure 2: Denaturing polyacrylamide gel showing the splicing pattern of Dscam exon 4 isoform variables after digestion with restricition enzymes 5 h and 10 h after injection of Thiamethoxam (1 μM) or the combination of Thiamethoxam, Carbendazim and Glyphosate (T+C+G, 1 μM/2 mM/47 mM). The undigested PCR is shown at the bottom. Ma: DNA marker

Fig 2 C

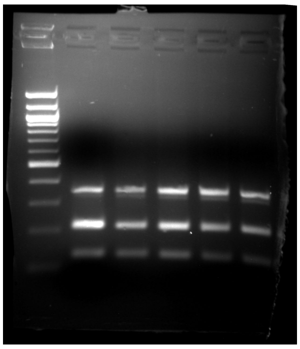

Fig 2 D gel used for quantification

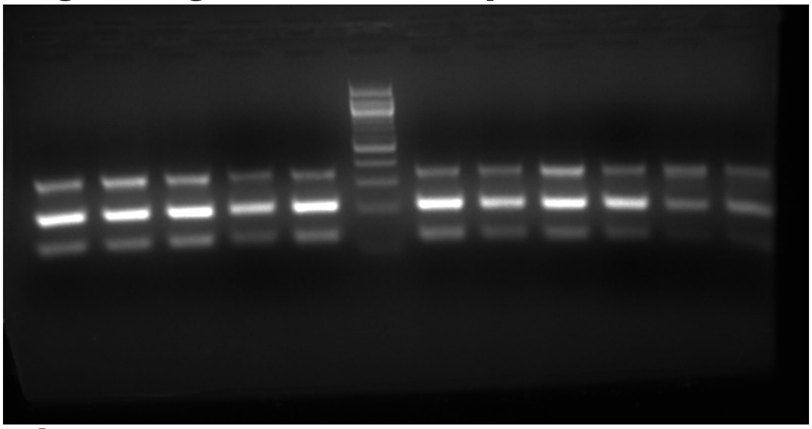

Fig 2 E

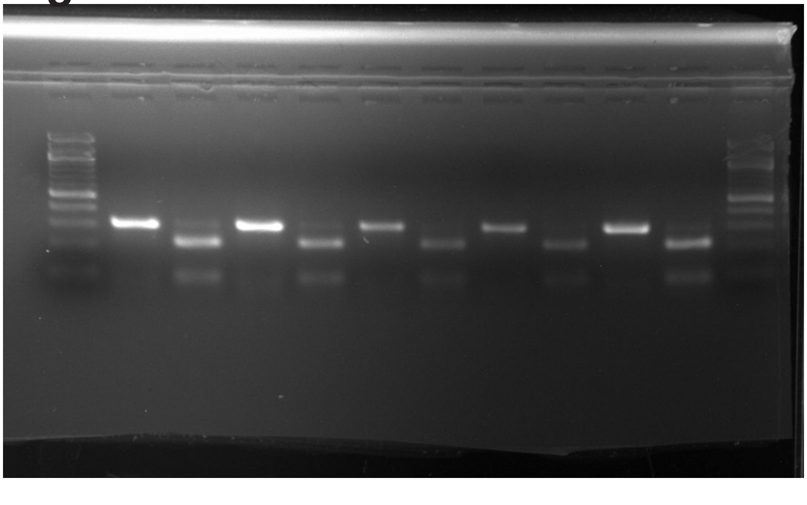

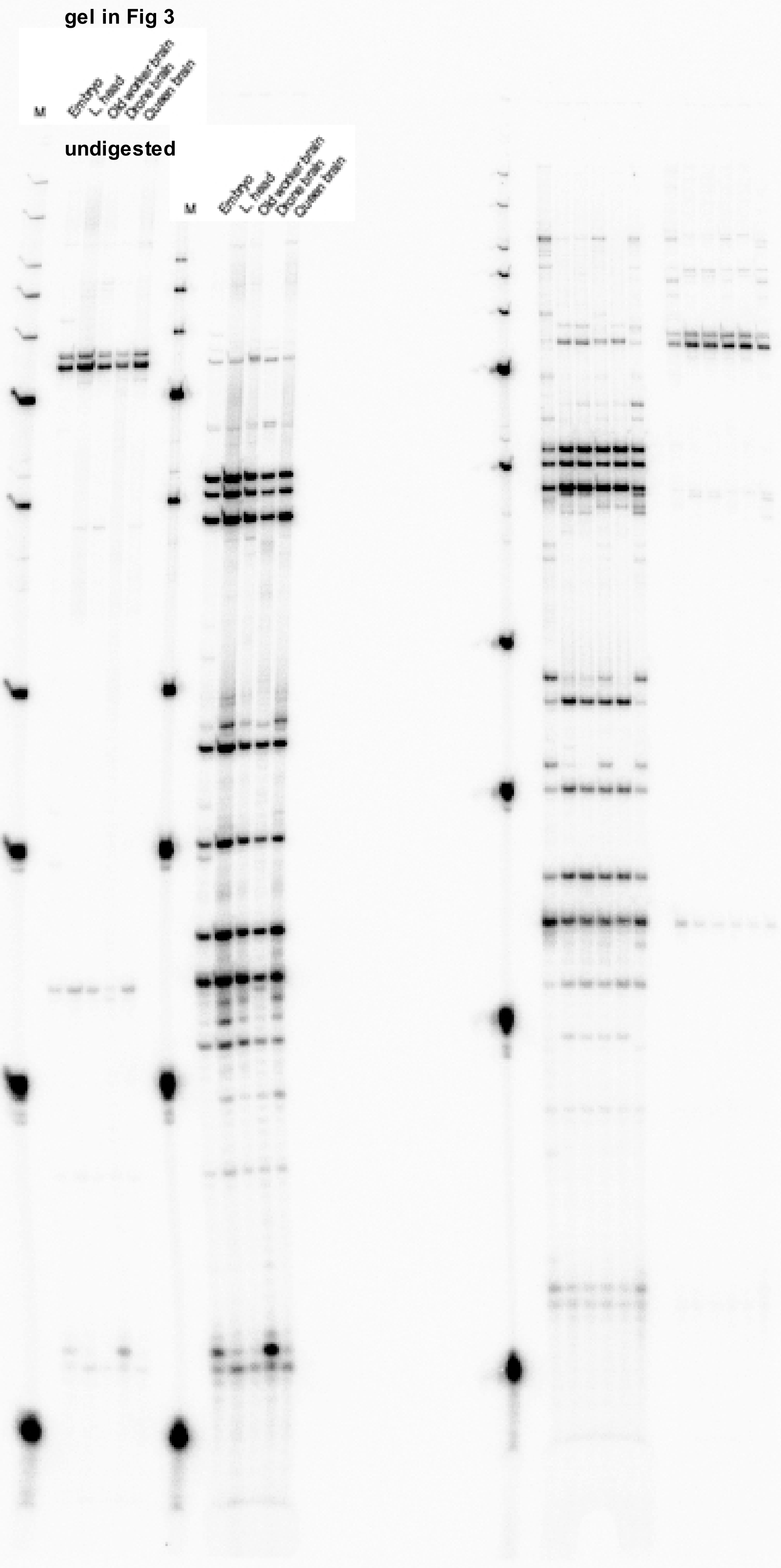
gel in Fig 3
M
Embryo
L. head
Old worker brain
Drone brain
Queen brain
undigested
M
Embryo
L. head
Old worker brain
Drone brain
Queen brain

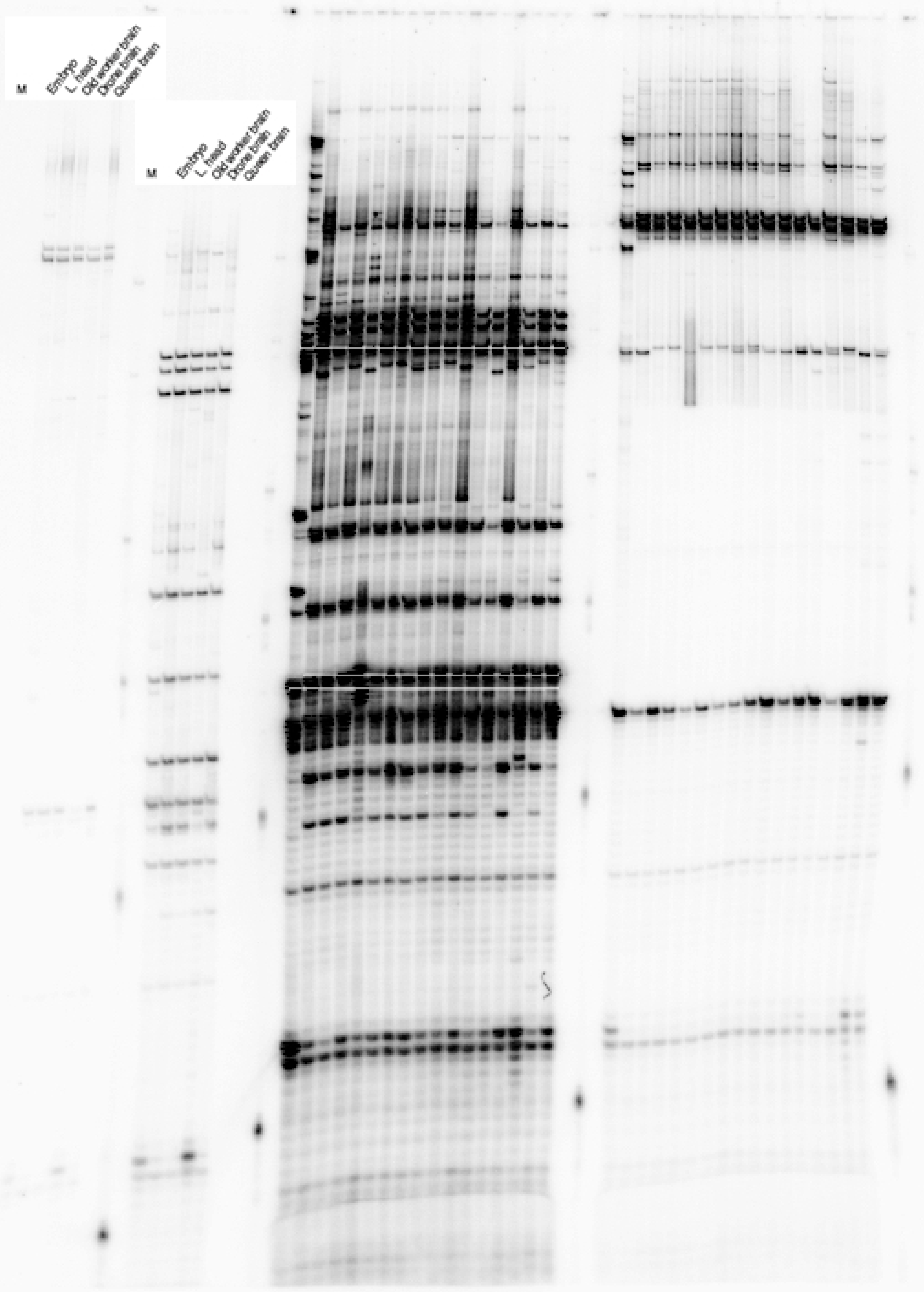

gel used for quantification in Fig 3

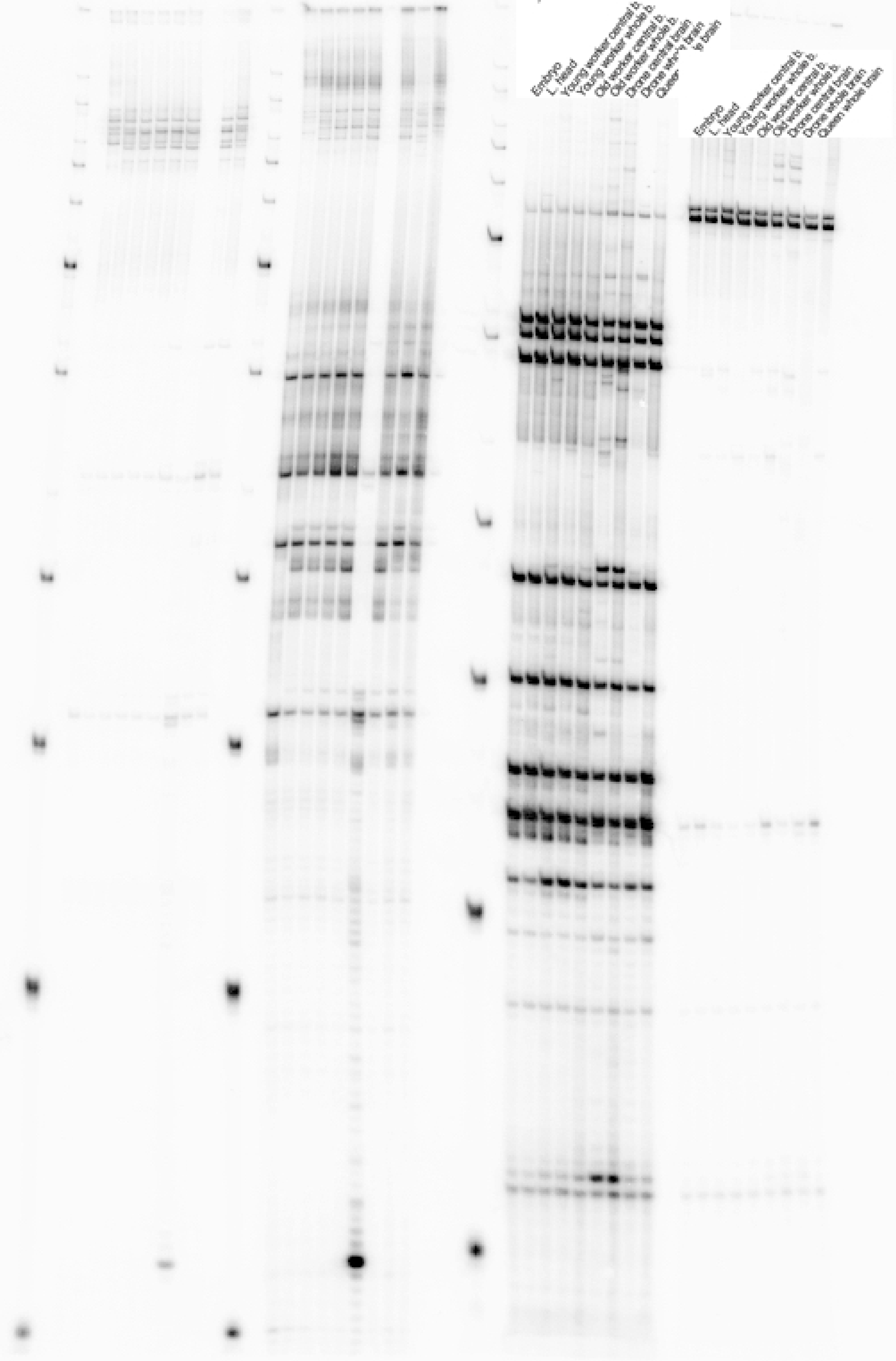

gel used for quantification in Fig 3

Fig 4

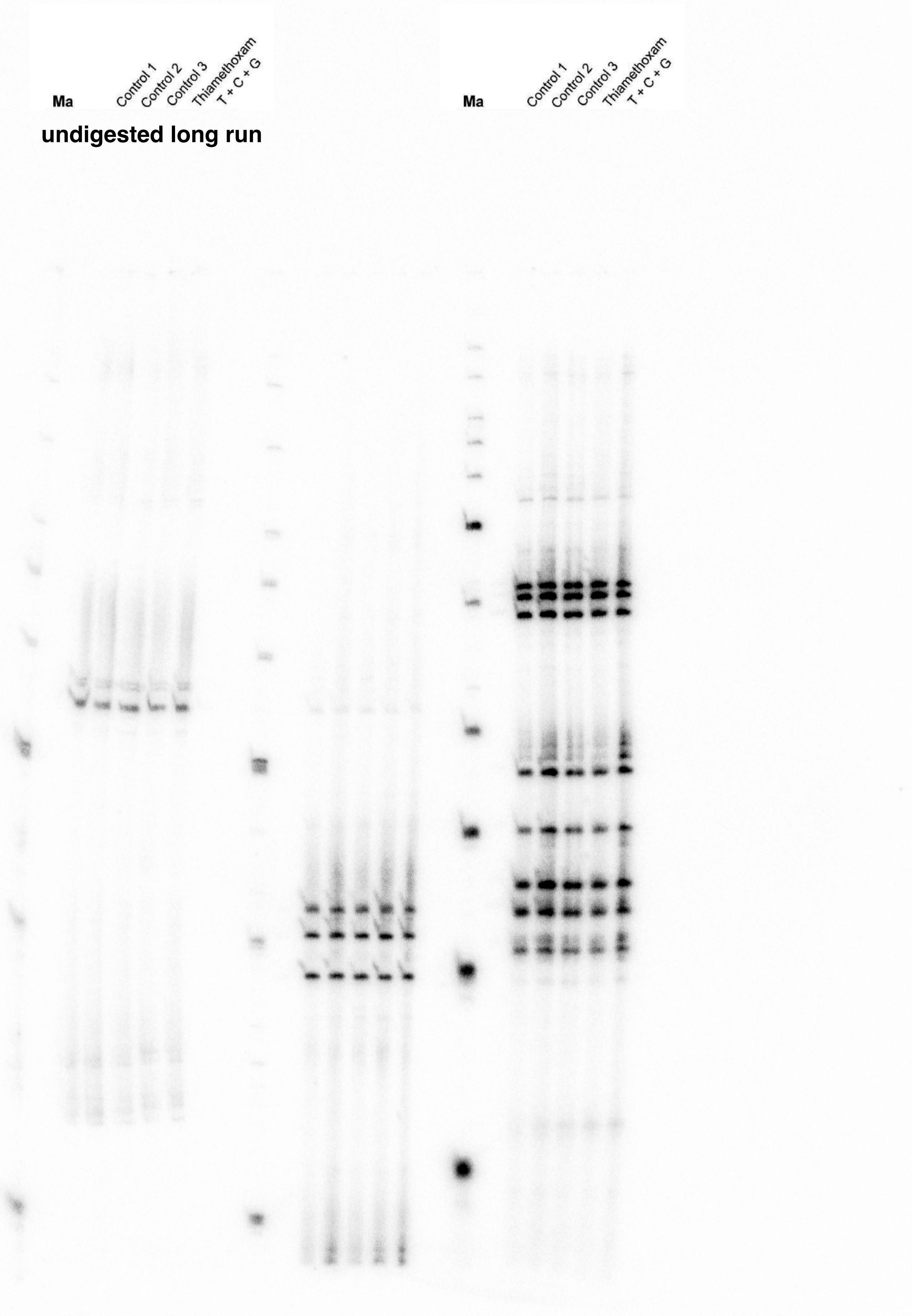
Ma
Control 1
Control 2
Control 3
Thiamethoxam
T + C + G
undigested long run
Ma
Control 1
Control 2
Control 3
Thiamethoxam
T + C + G

Fig 5

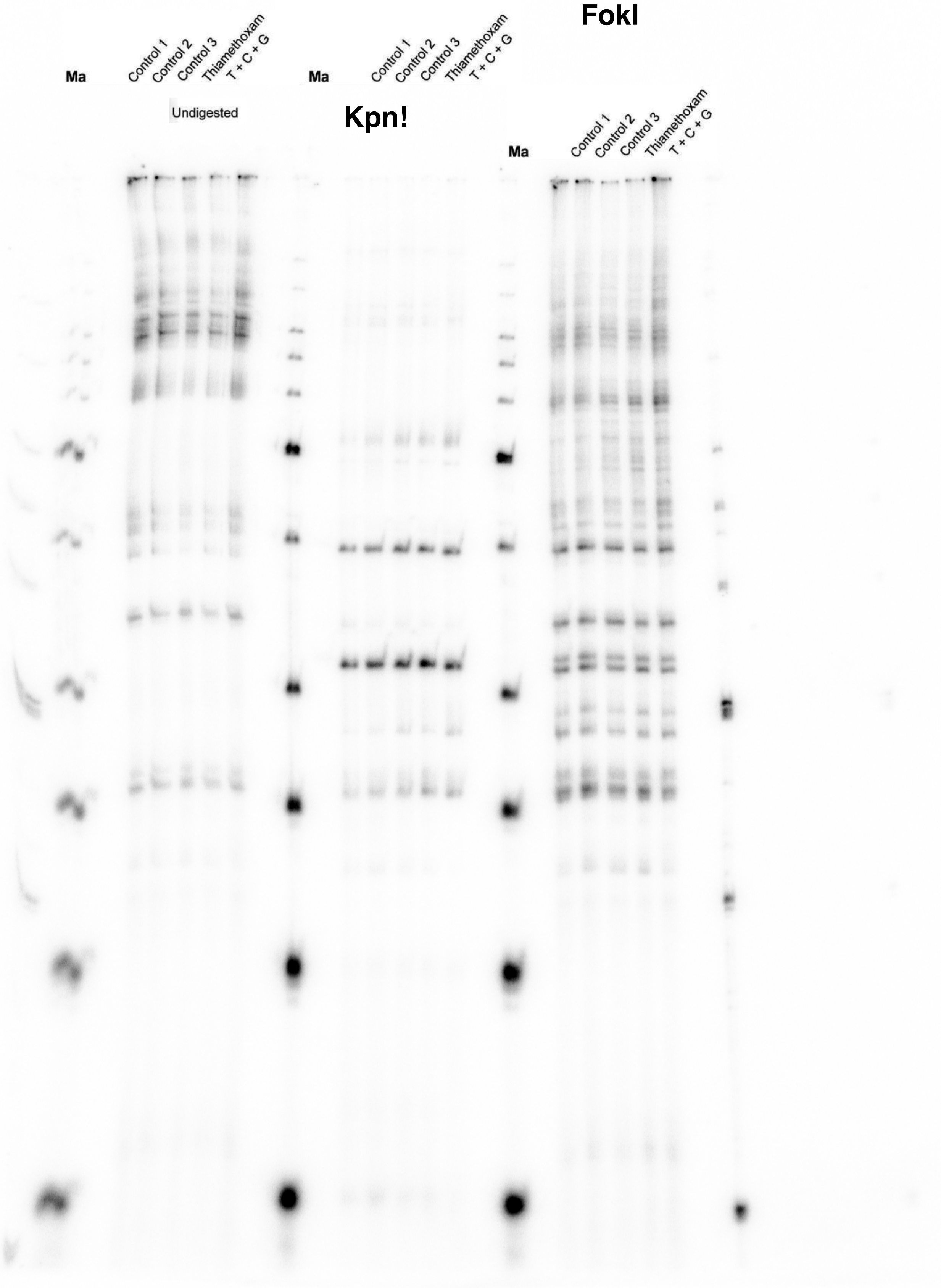
FokI
Ma
Control 1
Control 2
Control 3
Thiamethoxam
T + C + G
Ma
Control 1
Control 2
Control 3
Thiamethoxam
T + C + G
Undigested
Kpn!
Ma
Control 1
Control 2
Control 3
Thiamethoxam
T + C + G

Supplemental Figure 2
undigested

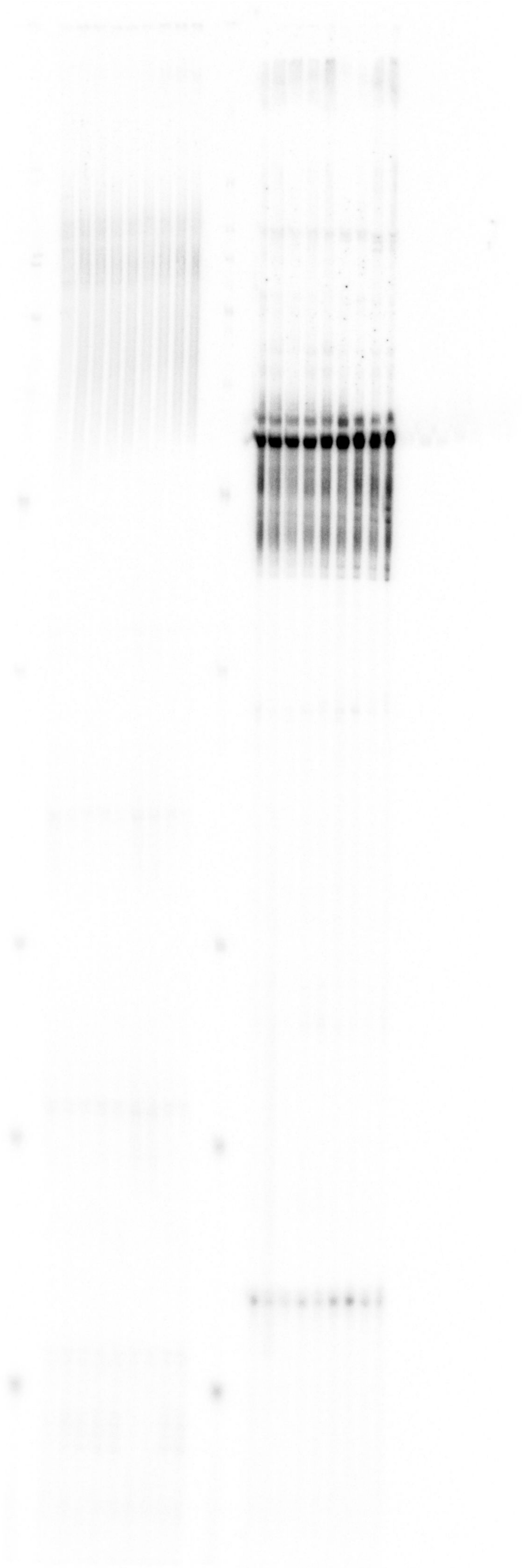

Supplemental Figure 2
digested

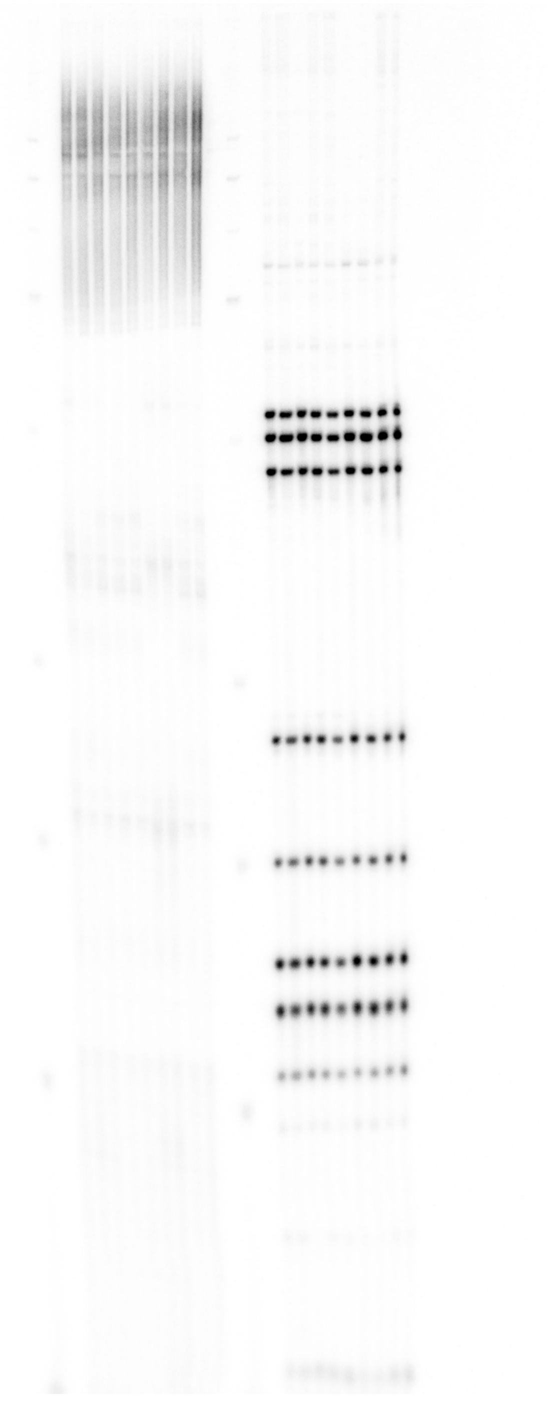

Supplement: Supplementary file 1 — Supplementary information [file 41598_2019_55534_MOESM1_ESM.pdf]
